# A Surgical Treatment of Locomotor Ataxia

**Published:** 1912-06

**Authors:** 


**A Surgical Treatment of Locomotor Ataxia.** By **L. N. Denslow,** M.D.; Bailliere, Tindall & Co., London, 1912, 12 mo., p.p. 118.

A very recent small work from the pen of a well known recent American scholar, with the above title, has just appeared in attractive form, with still more attractive subject matter. Men who have lived during the past generation as well as the present will well remember the unmistakable views enunciated by the late Dr. Fessenden Otis regarding strictures of large calibre, constrictions of the meatus, and the great variety of reflexes which might emanate from the urethra involved in either of these conditions. The application of the Otis dicta to the conditions summed up under the general name of locomotor ataxia is not necessarily new save to recent students, but in this concrete form will serve as a reminder of a matter now too generally forgotten in the hurry and haste with which opinions chase each other in recent times. Dr. Denslow has done a great service by connecting up the phenomena of the latter condition with the pathology of the urethra as enunciated by Otis. It will not do to say that urethral conditions are the secret mainspring of all spinal scleroses, or other lesions, but the fact remains that men have become oblivious to the diversified reflexes of urethral origin. Otis was distinct and unmistakable in his teachings. Their value was probably never fully appreciated, and is today generally forgotten. The gist of this very valuable little book consists in this, that after a brief summary of the clinical features and the pathologic conditions met with in many cases of locomotor ataxia, it is shown how many of them may be, and probably are due to previously underlying and, perhaps, forgotten urethral lesions. Behind all this matter is a principle of the greatest value, i.e., not alone the clinical features of ataxy may be due to such local disturbances but many others which the author does not attempt to consider. To one who has followed, in time past, the teachings of Otis, and who well remembers the controversy between himself and the late Dr. Sands, this book will induce a revival of interest and a collection of reminiscences of really great and enduring importance. It is Denslow's especial intent to show how often those lesions generally comprehended and included under the term "locomotor ataxia" may be distinctly ascribed to a direct relation to narrowings somewhere in the urethral canal In view of what has been known and partly forgotten and, especially, in view of that of which sight should never be lost, his suggestions as to careful examination of the urethra in order to note its varying diameters or calibres, and to remedy all faults of the latter, are of the greatest clinical import and importance. The reviewer, therefore, considers this little book of Dr. Denslow's as an exceedingly valuable contribution to the therapy and consideration of a disease hitherto too generally considered progressive, unpromising, and usually helpless, and he sides entirely with the author in urging systematic investigation, in this direction, of every patient suffering from any spinal malady which can, or even perhaps cannot be, grouped under the general title. The brochure is well worthy of careful reading, and its suggestions well worth carefully trying, as offering greater promise of helpfulness than has yet been afforded from any other method of treatment. R. P.

